# Photorespiration Coupled With CO_2_ Assimilation Protects Photosystem I From Photoinhibition Under Moderate Poly(Ethylene Glycol)-Induced Osmotic Stress in Rice

**DOI:** 10.3389/fpls.2020.01121

**Published:** 2020-07-24

**Authors:** Shinya Wada, Chikahiro Miyake, Amane Makino, Yuji Suzuki

**Affiliations:** ^1^ Faculty of Agriculture, Iwate University, Morioka, Japan; ^2^ Graduate School of Agricultural Science, Kobe University, Nada-ku, Japan; ^3^ Graduate School of Agricultural Science, Tohoku University, Aoba-ku, Japan

**Keywords:** osmotic stress, Rubisco, photorespiration, CO_2_ assimilation, photosystem I, photosystem II, rice

## Abstract

Photorespiration coupled with CO_2_ assimilation is thought to act as a defense system against photoinhibition caused by osmotic stress. In the present study, we examined whether such a mechanism is operative for the protection of photosystem I (PSI) in rice (*Oryza sativa* L.) including transgenic plants with decreased and increased Rubisco content (*RBCS*-antisense and *RBCS*-sense plants, respectively). All plants were hydroponically grown and moderate osmotic stress was imposed using hydroponic culture solutions containing poly(ethylene glycol) (PEG) at 16% or 20% (w/v) for 2 d. In wild-type plants, the rates of CO_2_ assimilation (*A*) were significantly decreased by the PEG treatment, whereas the photorespiration activity estimated from the rates of electron transport in photosystem II (PSII) and *A* were not affected. The maximal quantum efficiency of PSII (*F*_v_/*F*_m_) and the maximal activity of PSI (*P*_m_) were also not affected. In *RBCS*-antisense plants, *A* and the estimated photorespiration activity were considerably lower than those in wild-type plants in the presence or absence of the PEG treatment. *P*_m_ and both *F*_v_/*F*_m_ and *P*_m_ decreased in the 16% PEG-treated and 20% PEG-treated *RBCS*-antisense plants, respectively. Thus, the decrease in Rubisco content led to the photoinhibition of PSI and PSII, indicating the importance of photorespiration coupled with CO_2_ assimilation for the protection of PSI from moderate PEG-induced osmotic stress. It was also shown that PSI was more sensitive to osmotic stress than PSII. In the PEG-treated wild-type and *RBCS*-antisense plants, osmotic-stress responses of the photosynthetic electron transport reactions upstream of PSI led to the oxidation of P700, which is thought to prevent PSI from over-reduction. Although such a defense system operated, it was not sufficient for the protection of PSI in *RBCS*-antisense plants. In addition, there were no large differences in the parameters measured between wild-type and *RBCS*-sense plants, as overproduction of Rubisco did not increase photorespiration activity.

## Introduction

Drought stress is one of the most harmful environmental stresses on plant productivity. Stomatal closure in response to drought stress prevents water loss *via* transpiration but decreases CO_2_ availability within a leaf and energy consumption by the Calvin-Benson cycle (Lawlor and Tezara, 2009). The resulting excess light energy can over-reduce the photosynthetic electron transport (PET) chain (Cruz de Carvalho, 2008; Xu et al., 2010) and generate reactive oxygen species (ROS) around photosystem II and I (PSII and PSI, respectively) (Asada, 1999; Müller et al., 2001; Krieger-Liszkay, 2005), leading to photoinhibition of these photosystems. PSI photoinhibition requires a long period of recovery and severely decreases photosynthesis and plant growth (Kudoh and Sonoike, 2002; Sonoike, 2011), whereas PSII photoinhibition is repaired efficiently in a short period of time (Demmig-Adams and Adams, 1992; Murata et al., 2007). It has been observed that PSI suffered from photoinhibition under severe drought stress, whereas PSII was not largely affected in some tropical tree species (Huang et al., 2013). Similar phenomena were observed when rice plants were subjected PEG-induced osmotic stress, which are widely used to mimic drought stress (Wada et al., 2019). These results show that PSI is more sensitive to drought or osmotic stress than PSII. Therefore, PSI photoinhibition would be harmful under such stress conditions.

It has been reported that the PET reactions responded to drought or osmotic stress in a manner that limits the electron flow toward PSI. Such responses include the non-photochemical quenching (NPQ) of light energy at PSII (Golding and Johnson, 2003; Zhou et al., 2007; Lawlor and Tezara, 2009; Huang et al., 2012; Zivcak et al., 2013; Zivcak et al., 2014; Wada et al., 2019) and limitation of the electron flow at the cytochrome *b*_6_/*f* complex (Kohzuma et al., 2009). These events were accompanied by the oxidation of the reaction center chlorophyll of PSI, P700 (Golding and Johnson, 2003; Huang et al., 2012; Zivcak et al., 2013; Zivcak et al., 2014; Wada et al., 2019), which was suggested to suppress the production of ROS in PSI (Sejima et al., 2014; Takagi et al., 2017a). These results strongly suggest that these drought- or osmotic-stress responses of the PET reactions protect PSI from over-reduction and photoinhibition by ROS.

In addition to these responses of the PET reactions, processes downstream of PSI can also contribute to the protection of PSI under drought stress. One such process is photorespiration, a large and energy-consuming pathway that salvages byproducts of the reaction of Rubisco in the Calvin-Benson cycle (Ogren, 1984). Rubisco catalyzes not only the carboxylation of ribulose 1,5-bisphosphate, which generates two molecules of 3-phosphoglycerate for CO_2_ assimilation, but also its oxygenation, which generates one molecule each of 2-phosphoglycolate and 3-phosphoglycerate. The photorespiratory pathway converts 2-phosphoglycolate to 3-phosphoglycerate while consuming reducing equivalents and ATP. Rubisco oxygenase activity and photorespiration are relatively active under CO_2_-limited conditions according to the C3 photosynthesis model of Farquhar and co-workers (Farquhar et al., 1980; von Caemmerer, 2000). It was suggested that the rate of CO_2_ and O_2_ uptake by carboxylation and oxygenation reactions, respectively, is at the ratio of 1:2 under the CO_2_ compensation point, and that the Calvin-Benson cycle and the photorespiratory pathway operate in a balanced state. Photorespiration was estimated to consume a large portion of light energy under such conditions (Sejima et al., 2016; Hanawa et al., 2017). The rates of energy consumption by photorespiration were reported to increase in response to drought or osmotic stress (Cornic and Briantais, 1991; Wingler et al., 1999; Haupt-Herting and Fock, 2002; Galmés et al., 2007; Zivcak et al., 2013; Chastain et al., 2014; Wada et al., 2019). It was also found that drought-stress induced NPQ, and that NPQ was further stimulated in barley mutants with decreased activity of a photorespiratory enzyme, suggesting that photorespiration consumes excess light energy under drought stress (Wingler et al., 1999).

However, it remains unclear whether photorespiration coupled with CO_2_ assimilation protects PSI under drought or osmotic stress. In the present study, this was explored in transgenic rice (*Oryza sativa* L.) plants with decreased Rubisco content (*RBCS*-antisense plants; Makino et al., 2000). We have recently reported that the PET chain was over-reduced in *RBCS*-antisense plants under the combination of high irradiance and CO_2_-compensated conditions (Wada et al., 2018). PSI also became susceptible to excess light energy imposed by repetitive illumination of saturated pulse-light, which is thought to generate ROS in PSI (Sejima et al., 2014; Zivcak et al., 2015). Transgenic rice plants with increased Rubisco content (*RBCS*-8sense plants; Suzuki et al., 2007) were also used as control plants. We have previously observed that the activities of photorespiration and CO_2_ assimilation were not substantially enhanced in *RBCS*-sense plants (Makino and Sage, 2007; Suzuki et al., 2007; Suzuki et al., 2009; Wada et al., 2018). Plants were exposed to moderate osmotic-stress treatments using poly(ethylene glycol) (PEG)-containing culture solutions. The maximal quantum efficiency of PSII (*F*_v_/*F*_m_) and the maximal P700 signal of PSI (*P*_m_) were determined as indices of photoinhibition and are discussed in relation to the activities of photorespiration and CO_2_ assimilation. In addition, osmotic-stress responses of the PET reactions were also examined by measuring chlorophyll fluorescence and P700 absorbance and its relationship with the activities of photorespiration and CO_2_ assimilation are discussed.

## Materials and Methods

### Plant Culture

Rice (*Oryza sativa* L. “Notohikari”) plants were used as wild-type plants and the background cultivar for the previously generated Rubisco-transgenic plants. T_4_ progenies of *RBCS*-antisense plants (line AS-71; Makino et al., 2000) and BC_2_ progenies of *RBCS*-sense plants (line Sr-26-8; Suzuki et al., 2007) were used. Each plant was grown hydroponically in a growth chamber (NC-441HC, NKsystem, Osaka, Japan) operated under the conditions of photon flux density of 400–500 μmol photon m^−2^ s^−1^, a photoperiod of 14 h, and day/night temperature regime of 27/22°C. Pre-soaked seeds were sown and germinated on a net floating on tap water, whose pH was adjusted to 5.3–5.5 with 1 M HCl. After 2 weeks, seedlings were transplanted into 1.1 L plastic pots filled with the culture solution. The composition of the culture solution is described in Makino et al. (1988). The culture solution was renewed once a week. The concentration of the culture solution was increased depending on plant growth.

### Osmotic-Stress Treatments Using PEG

Plants grown for approximately 60 d after sowing were subjected to osmotic stress treatments using PEG with an average molecular weight of 6,000 (PEG, Sigma-Aldrich, St. Louis, MO, USA). The culture solution containing PEG at the concentration of 16 or 20% (w/v) was supplied instead of the regular culture solution for 2 d in the growth chamber described above. After the treatments, the uppermost, fully expanded leaves were used for the measurement of photosynthesis and biochemical assays.

### Measurements of Photosynthesis

The rate of CO_2_ assimilation (*A*), chlorophyll fluorescence, and P700 absorbance were simultaneously measured using the combination system of GFS-3000 and DUAL-PAM-100 (Heinz Walz GmbH, Effeltrich, Germany). The detailed conditions are described in Wada et al. (2019). Briefly, *F*_v_/*F*_m_ and *P*_m_ were measured after the leaves were dark-adapted, followed by the measurements of chlorophyll fluorescence and P700 absorbance under the conditions of an actinic light intensity of 1,200 μmol photon m^−2^ s^−1^, an ambient CO_2_ partial pressure of 40 Pa, a leaf temperature of 27°C, and a relative humidity of 60–70%. The quantum efficiency of PSII [Y(II)], the quantum yields of the NPQ [Y(NPQ)] and of the non-regulated and non-photochemical energy dissipation [Y(NO)], and the index for the reduction of the primary plastoquinone electron acceptor in PSII (Q_A_) (1-q_L_) were calculated following the methods described by Kramer et al. (2004) and Baker (2008). Three complementary quantum yields were defined: Y(II) + Y(NO) + Y(NPQ) = 1. The rate of electron transport in PSII (ETRII) was calculated as Y(II) × photon flux density × α × 0.5. The absorptance (α) was adopted to be 0.84 in this study. The quantum efficiency of PSI [Y(I)] and the quantum yields of the donor side limitation of PSI [Y(ND)] and of the acceptor side limitation of PSI [Y(NA)] were calculated according to the methods described by Klughammer and Schreiber (1994) and Schreiber and Klughammer (2008). Three complementary quantum yields were defined: Y(I) + Y(NA) + Y(ND) = 1. The rate of electron flow donated for photorespiration (*J*_PR_) was evaluated using the equation of *J*_PR_ = 2/3 × [ETRII – 4 (*A* + *R*_d_)] (Valentini et al., 1995; Zivcak et al., 2013). *R*_d_ was the rate of respiration under illumination and was assumed to be 1 μmol m^−2^ s^−1^ as in our previous study (Suzuki et al., 2007).

### Measurements of the Relative Water Content of Leaves

The relative water content of the leaves (RWC) was determined after the stress treatment, following the methods of Zhou et al. (2007), as described in Wada et al. (2019), using leaf fresh weight measured just after the stress treatment, leaf weight after overnight immersion in deionized water at 4°C, and leaf dry weight.

### Biochemical Assays

Leaves were collected after the measurement of photosynthesis, frozen using liquid nitrogen, and kept at −80°C until use. Total leaf-N, chlorophyll, and Rubisco content were determined as described in Makino et al. (1997). Briefly, total leaf-N content was determined using Nessler’s reagent after Kjeldahl digestion. Arnon’s method (Arnon, 1949) was used for chlorophyll determination. Rubisco content was determined by formamide extraction of Coomassie Brilliant Blue R-250-stained bands corresponding to the large and small subunits of Rubisco separated by SDS-PAGE (Makino et al., 1985), except that bovine serum albumin was used to prepare the calibration curves.

### Statistical Analysis

Three to five biological replicates were analyzed using the Tukey-Kramer’s HSD test using JMP 14 (SAS Institute Japan, Tokyo, Japan). The Pearson correlation coefficients of the measured parameters were calculated using Microsoft Excel 2013.

## Results

**Table 1** shows the amounts of Rubisco protein, chlorophyll, and total leaf-N in leaves of the PEG-untreated wild-type, *RBCS*-sense, and *RBCS*-antisense plants. The amounts of Rubisco in *RBCS*-sense and *RBCS*-antisense plants were 120% and 43%, respectively, of the levels in the wild-type plants. The amount of chlorophyll in the *RBCS*-sense plants tended to be slightly lower than that in wild-type plants, whereas the amount of total leaf-N was not different. In *RBCS*-antisense plants, the amounts of chlorophyll and total leaf-N were lower than those in wild-type plants. Such trend was also observed previously (Makino and Sage, 2007; Suganami et al., 2018; Wada et al., 2018). The magnitude of these changes was smaller than that in the amount of Rubisco. Thus, the amounts of Rubisco were greatly affected by genetic manipulation.

**Table 1:** Amounts of Rubisco protein, chlorophyll, and total leaf-nitrogen in the uppermost, fully expanded leaves in wild-type, *RBCS*-sense, and *RBCS*-antisense rice plants.

	Rubisco (g m^−2^)	Chlorophyll (mmol m^−2^)	Total leaf-N (mmol m^−2^)
Wild-type	3.45 ± 0.13^B^ (100)	0.78 ± 0.04^A^	142.3 ± 5.2^A^
*RBCS*-sense	4.13 ± 0.23^A^ (120)	0.71 ± 0.03^AB^	140.9 ± 4.6^A^
*RBCS*-antisense	1.47 ± 0.03^C^ (43)	0.63 ± 0.02^B^	104.2 ± 1.9^B^

The relative amount of Rubisco when the wild-type level was defined as 100 is shown in parentheses. Data are presented as means ± SE (n = 4). Statistical analysis was carried out using ANOVA followed by the Tukey–Kramer’s test. Columns with the same letter are not significantly different (p < 0.05).

**Figure 1:**
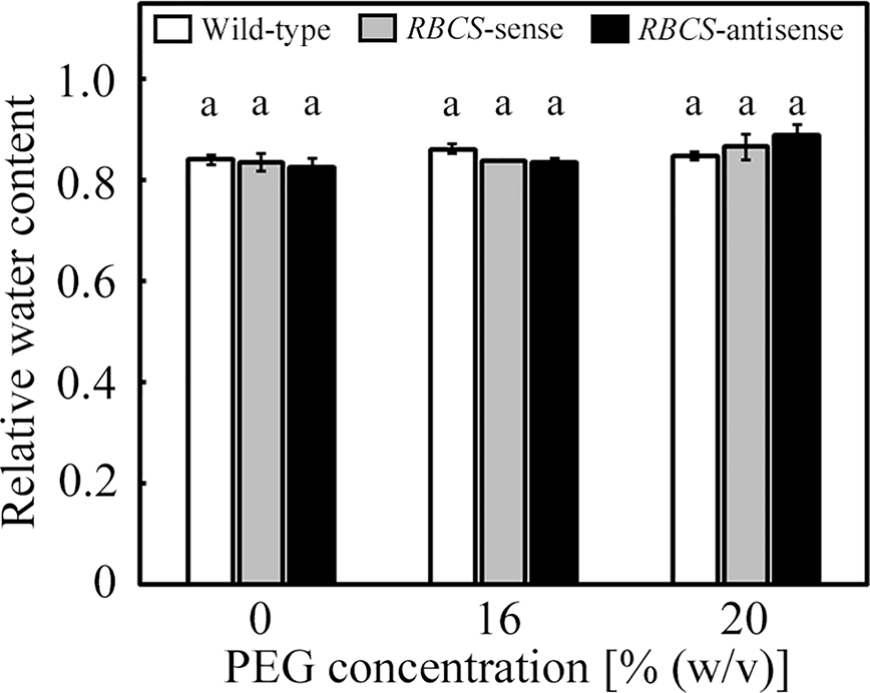
Relative water content of leaves after water stress treatment in transgenic rice plants with an increased (*RBCS*-sense) or decreased (*RBCS*-antisense) Rubisco content. Wild-type plants were used as a control. Sixty days after germination, hydroponically grown plants were water-stressed using culture solutions containing PEG at 0, 16, and 20% (w/v) for 2 d under an irradiance of 400–500 μmol photon m^−2^ s^−1^ and day/night air-temperatures of 27/22°C, followed by the measurements of relative water content of leaves. White, gray, and black bars denote wild-type, *RBCS*-sense, and *RBCS*-antisense plants, respectively. Data are presented as average ± standard error (*n* = 3–4). Statistical analysis was carried out using ANOVA with a post-hoc Tukey’s HSD test. Columns with the same letter are not significantly different (*p* < 0.05). Statistically significant differences were not observed.

The culture solutions containing PEG at concentrations of 16 and 20% (w/v) were used to impose osmotic stress to the plants. We have previously observed that values of the relative water content of leaves of wild-type rice plants only marginally decreased under these PEG treatments (Wada et al., 2019). In the present study, the relative water content of leaves was not significantly affected by the PEG treatments, and did not significantly differ among genotypes (**Figure 1**).

**Figure 2:**
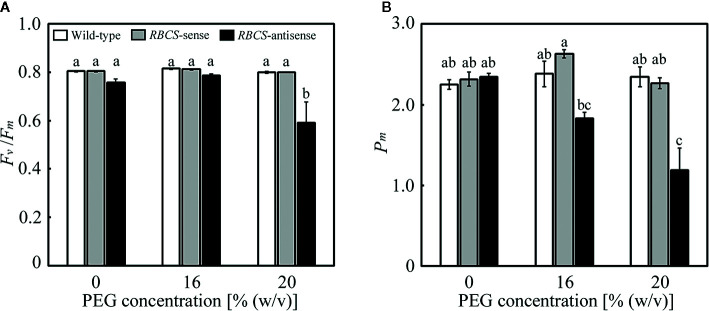
Maximum quantum efficiency of PSII photochemistry (*F*_v_/*F*_m_) **(A)** and the maximal P700 signal (*P*_m_) **(B)** after water stress treatment in transgenic rice plants with an increased (*RBCS*-sense) or decreased (*RBCS*-antisense) Rubisco content. Wild-type plants were used as a control. Sixty days after germination, hydroponically grown plants were water-stressed using culture solutions containing PEG at 0, 16, and 20% (w/v) for 2 d under an irradiance of 400–500 μmol photon m^−2^ s^−1^ and day/night air-temperatures of 27/22°C, followed by the measurements of *F*_v_/*F*_m_ and *P*_m_. White, gray, and black bars denote wild-type, *RBCS*-sense, and *RBCS*-antisense plants, respectively. Data are presented as average ± standard error (*n* = 4–5). Statistical analysis was carried out using ANOVA with a post-hoc Tukey’s HSD test. Columns with the same letter are not significantly different (*p* < 0.05).

Effects of the PEG treatments on the fitness of the photosynthetic system were evaluated using *F*_v_/*F*_m_ and *P*_m_, which are the indices of the photoinhibition of PSII and PSI, respectively. It has been previously shown that *F*_v_/*F*_m_ and *P*_m_ were not affected under these PEG treatments in wild-type rice plants. In the PEG-untreated plants, there were no differences in *F*_v_/*F*_m_ and *P*_m_ between wild-type and *RBCS*-sense plants (**Figure 2**). There was no statistical difference between wild-type plants and *RBCS*-antisense plants, although *F*_v_/*F*_m_ and *P*_m_ in the latter tended to be marginally lower. Similar trend has been observed in *RBCS*-antisense plants previously (Hirotsu et al., 2004). Neither *F*_v_/*F*_m_ nor *P*_m_ changed in the PEG-treated wild-type and *RBCS*-sense plants, indicating that these genotypes did not suffer from the photoinhibition of PSII or PSI. In contrast, *F*_v_/*F*_m_ substantially decreased to 0.59 in the 20% PEG-treated *RBCS*-antisense plants (**Figure 2A**). *P*_m_ in the 16% PEG-treated plants decreased to 78% of the level of the PEG-untreated *RBCS*-antisense plants, and further decreased to 51% in the 20% PEG-treated plants (**Figure 2B**). These results indicate that PSI and both PSII and PSI underwent photoinhibition in *RBCS*-antisense plants under the 16%- and 20%-PEG treatments, respectively. It is also indicated that PSI in *RBCS*-antisense plants was more sensitive to the PEG treatments than PSII.

**Figure 3:**
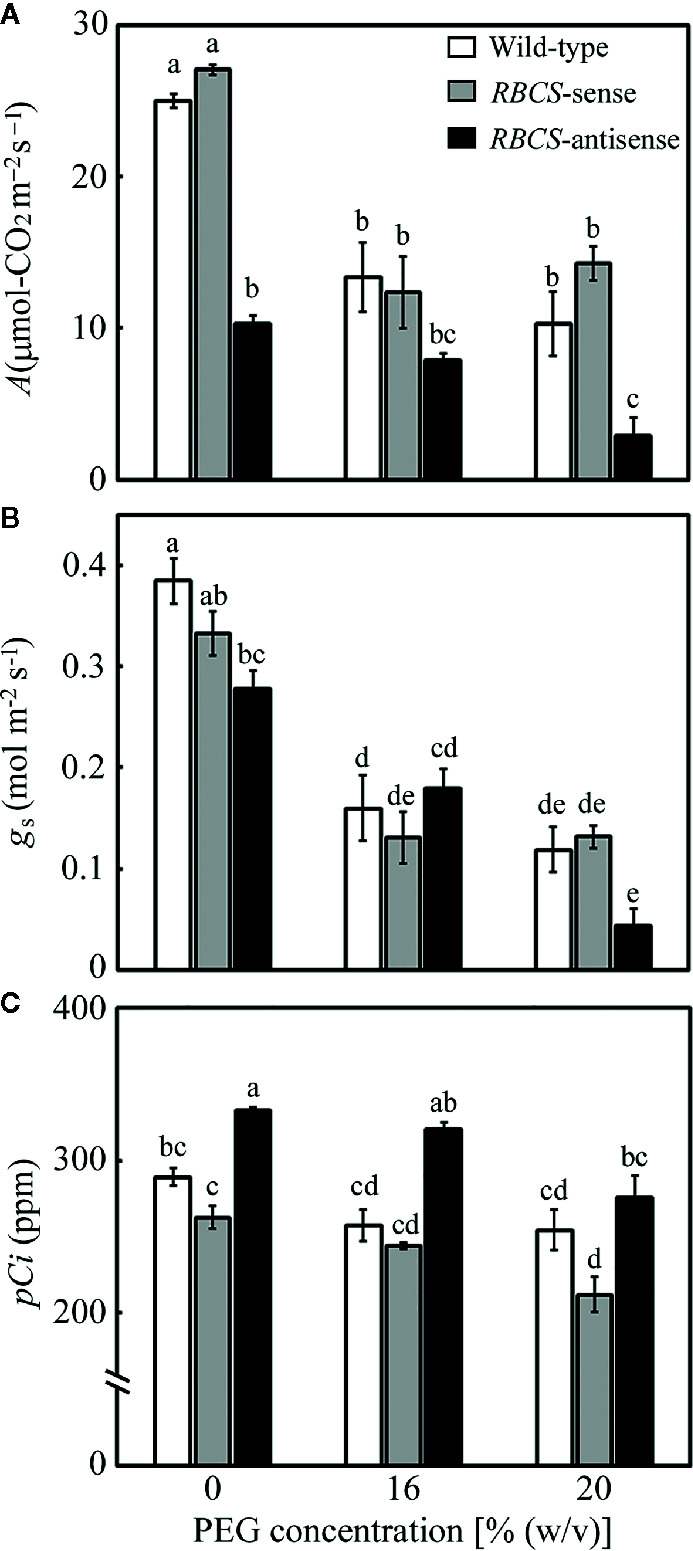
Rate of CO_2_ assimilation (*A*) **(A)**, stomatal conductance (*g*_s_) **(B)**, and intercellular CO_2_ partial pressure (*pCi*) **(C)** after water stress treatment in transgenic rice plants with an increased (*RBCS*-sense) or decreased (*RBCS*-antisense) Rubisco content. Wild-type plants were used as a control. Sixty days after germination, hydroponically grown plants were water-stressed using culture solutions containing PEG at 0, 16, and 20% (w/v) for 2 d under an irradiance of 400–500 μmol photon m^−2^ s^−1^ and day/night air-temperatures of 27/22°C. *A*, *g*_s_, and *pCi* were measured under the conditions of an actinic light intensity of 1,200 μmol photon m^−2^ s^−1^, an ambient CO_2_ partial pressure of 40 Pa, leaf temperature of 27°C, and relative humidity of 60–70%. White, gray, and black bars denote wild-type, *RBCS*-sense, and *RBCS*-antisense plants, respectively. Data are presented as average ± standard error (*n* = 4–5). Statistical analysis was carried out using ANOVA followed by the Tukey–Kramer’s test. Columns with the same letter are not significantly different (*p* < 0.05).

Changes in leaf gas-exchange parameters were examined (**Figure 3**). In all genotypes, *A*, stomatal conductance (*g*_s_), and intercellular CO_2_ partial pressure (*pCi*) tended to decrease in the PEG-treated plants. Although the relative water content in leaves was not affected (**Figure 1**), the PEG treatment was shown to lead to partial stomatal closure and concomitant changes in the leaf gas-exchange parameters. In wild-type plants, *A* in the PEG-treated plants decreased to 41–53% of the levels in the PEG-untreated control plants (**Figure 3A**). Similar trends were observed in *g*_s_. The values of *pCi* decreased by more than 30 ppm in the PEG-treated wild-type plants. The decreases in *pCi* were not as much as the decrease in both *A* and *g*_s_ (**Figure 2C**; Lawlor and Tezara, 2009). The values of *A*, *g*_s_, and *pCi* in *RBCS*-sense plants were not largely different from those in wild-type plants irrespective of (PEG) in the culture solutions, although slight decreases in *g*_s_ or *pCi* were observed in some cases (**Figures 3A–C**). In contrast, *A* in *RBCS*-antisense plants was lower than in other genotypes (**Figure 3A**). When not treated with PEG, *A* was 41% that of the wild-type level, corresponding to the magnitude of decreases in the amount of Rubisco (**Table 1**). Decreases in *A* were primarily accounted for by decreases in Rubisco content as observed in our previous studies (Hirotsu et al., 2004; Makino and Sage, 2007; Suganami et al., 2018; Wada et al., 2018). Therefore, it was unlikely that *RBCS*-antisense plants were suffering from PSII photoinhibition that affected *A* despite of decreases in chlorophyll content and marginal decreases in *F*_v_/*F*_m_ (**Table 1** and **Figure 2**). Although the level of *g*_s_ was lower than that in the wild-type plants (**Figure 3B**), *pCi* was higher by 44 ppm owing to the greatly decreased *A* (**Figure 3C**). In the 16% and 20% PEG-treated *RBCS*-antisense plants, the values of *A* were 76% and 28%, respectively, that of the PEG-untreated *RBCS*-antisense plants. These values were 59% and 28% of those in the wild-type plants treated with the same (PEG), respectively. As *g*_s_ decreased in the PEG-treated *RBCS*-antisense plants (**Figure 3B**), the values of *pCi* decreased by 12 and 58 ppm in the 16% and 20% PEG-treated plants, respectively. The *pCi* in the PEG-treated *RBCS*-antisense plants was still higher than that in the wild-type plants treated with the same (PEG) (**Figure 3C**).

**Figure 4:**
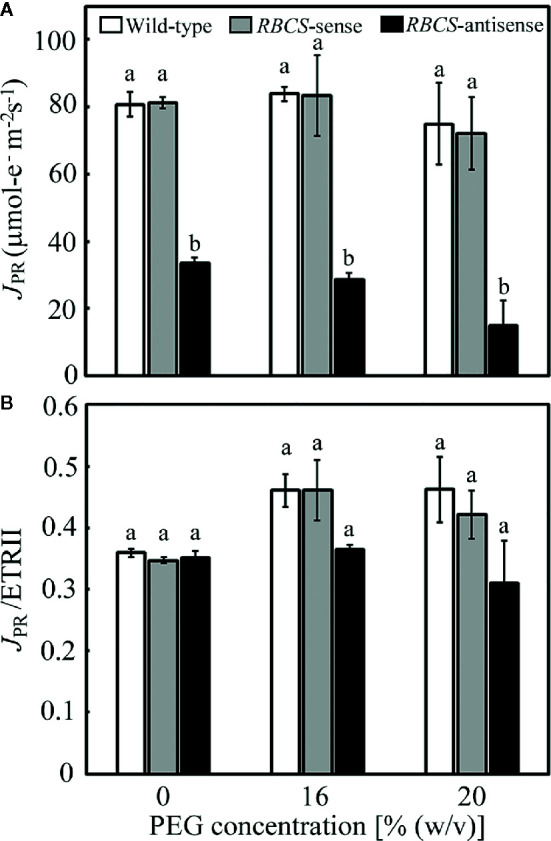
Rate of electron flow donated for photorespiration (*J*_PR_) **(A)** and its ratio to the electron transport rate in PSII (*J*_PR_/ETRII) **(B)** in transgenic rice plants with an increased (*RBCS*-sense) or decreased (*RBCS*-antisense) Rubisco content. Wild-type plants were used as a control. *J*_PR_ was evaluated with the equation *J*_PR_ = 2/3 × [ETRII – 4 (*A* + *R*_d_)] (Valentini et al., 1995; Zivcak et al., 2013). *R*_d_ was the rate of respiration under illumination and was assumed to be 1 μmol m^−2^ s^−1^ as in our previous study (Suzuki et al., 2007). ETRII was calculated as Y(II) × PFD × α × 0.5, where absorptance (α) was adopted to be 0.84. Data of *A* and Y(II) were taken from **Figures 3** and **5**, respectively. White, gray, and black bars denote wild-type, *RBCS*-sense, and *RBCS*-antisense plants, respectively. Data are presented as average ± standard error (*n* = 4–5). Statistical analysis was carried out using ANOVA followed by the Tukey–Kramer’s test. Columns with the same letter are not significantly different (*p* < 0.05).

The consumption of electrons by photorespiration, *J*_PR_, was calculated from *A* and ETRII (Valentini et al., 1995; Zivcak et al., 2013). The values of *J*_PR_ in the PEG-untreated wild-type plants and *RBCS*-sense plants were similar and did not change when treated by PEG (**Figure 4A**). In these genotypes, ratios of *J*_PR_ to ETRII were about 0.35 when not treated with PEG and tended to increase to 0.42–0.46 when treated with PEG (**Figure 4B**), indicating that the rate of consumption of electrons by photorespiration increased. *J*_PR_/ETRII was less than 0.5, showing that CO_2_ assimilation acted as a relatively greater electron sink, probably because stomata were still partially open and *pCi* was not greatly decreased under the present experimental conditions (**Figures 3B, C**). *J*_PR_ in *RBCS*-antisense plants was 41% of that in wild-type plants when not treated with PEG (**Figure 4A**). The magnitude of decreases in *J*_PR_ was similar to that in the amount of Rubisco (**Table 1**), as observed in the case of *A* (**Figure 3A**). *J*_PR_ further decreased in the 16% and 20% PEG treated *RBCS*-antisense plants. The values of *J*_PR_ in these plants corresponded to 34% and 20% of those in the wild-type plants treated with the same (PEG), respectively. These results show that the consumption of electrons by photorespiration and CO_2_ assimilation was greatly restricted owing to the decreased Rubisco content in *RBCS*-antisense plants. Ratios of *J*_PR_ to ETRII in *RBCS*-antisense plants were similar to those in wild-type plants when not treated with PEG (**Figure 4B**). In contrast to other genotypes, ratios of *J*_PR_ to ETRII in *RBCS*-antisense plants were relatively unchanged when treated with PEG, showing that the rate of consumption of electrons by photorespiration did not change.

**Figure 5:**
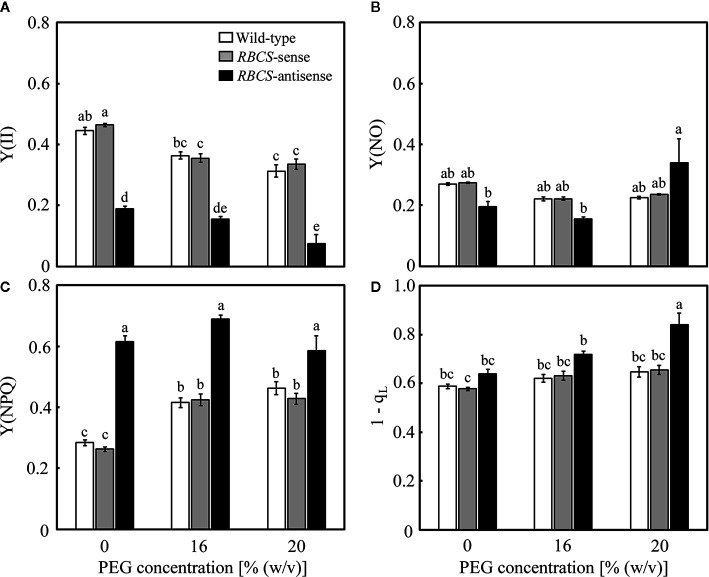
Chlorophyll fluorescence parameters after water stress treatment in transgenic rice plants with an increased (*RBCS*-sense) or decreased (*RBCS*-antisense) Rubisco content. Wild-type plants were used as a control. Sixty days after germination, hydroponically grown plants were water-stressed using culture solutions containing PEG at 0, 16, and 20% (w/v) for 2 d under an irradiance of 400–500 μmol photon m^−2^ s^−1^ and day/night air-temperatures of 27/22°C. Y(II) **(A)**, Y(NPQ) **(B)**, Y(NO) **(C)**, and 1−q_L_ **(D)** were measured under the conditions of an actinic light intensity of 1,200 μmol photon m^−2^ s^−1^, an ambient CO_2_ partial pressure of 40 Pa, leaf temperature of 27°C, and relative humidity of 60–70%. Data are presented as means ± SE (*n* = 4–5). Statistical analysis was carried out using ANOVA followed by the Tukey–Kramer’s test. Columns with the same letter are not significantly different (*p* < 0.05).

Changes in the photochemistry of PSII were examined in response to the PEG treatments. In wild-type plants, Y(II) decreased slightly and gradually as the (PEG) in the culture solution increased (**Figure 5A**). The magnitude of the decreases was smaller than that in *A* (**Figure 3A**). Slight decreases in Y(NO), which is an index for the dissipation of light energy in a non-regulated manner (Kramer et al., 2004), were also observed (**Figure 5C**). These changes were reflected in increases in Y(NPQ) (**Figure 5B**). The 1−q_L_ indicates the fraction of PSII centers in closed states (Kramer et al., 2004), which is thought to reflect the extent of the reduction of the plastoquinone pool (Miyake et al., 2009). The 1−q_L_ is also thought to be an index for lumenal acidification, as a decrease in q_L_ was accompanied by lumenal acidification in transgenic or transplastomic tobacco plants with decreases in the amounts of the chloroplastic ATP synthase (Rott et al., 2011). The values of 1−q_L_ tended to slightly increase in the PEG-treated wild-type plants (**Figure 5D**), suggesting the reduction of the plastoquinone pool and/or lumenal acidification. In *RBCS*-sense plants, the values of these parameters and their response to the PEG treatments were similar to those in wild-type plants (**Figures 5A–D**). In *RBCS*-antisense plants, Y(II) decreased to 43% of that in wild-type plants when not treated with PEG, while slight decreases in Y(NO) were also observed (**Figures 5A, C**). In contrast, Y(NPQ) increased to 2.2-fold higher than that in the PEG-untreated wild-type plants (**Figure 5B**), suggesting that light energy that became excessive because of the decrease in Rubisco content was dissipated primarily by NPQ (Hirotsu et al., 2004; Wada et al., 2018). At the same time, the values of 1−q_L_ tended to be higher than in other genotypes (**Figure 5D**). Y(II) further decreased in the PEG-treated *RBCS*-antisense plants and was lower than that in the wild-type plants treated with the same (PEG) (**Figure 4A**). In the 16% PEG-treated plants, a decrease in Y(NO) and an increase in Y(NPQ) were observed as in other genotypes (**Figures 5B, C**). In the 20% PEG-treated plants, a decrease in Y(II) was not accompanied by an increase in Y(NPQ) but by a substantial increase in Y(NO). Similar phenomena were observed in severely osmotic-stressed rice plants under high temperature (Wada et al., 2019). In addition, the values of 1−q_L_ increased in the PEG-treated *RBCS*-antisense plants and were higher than those in other genotypes treated with the same (PEG), suggesting that reduction of the plastoquinone pool and/or lumenal acidification was further enhanced.

**Figure 6:**
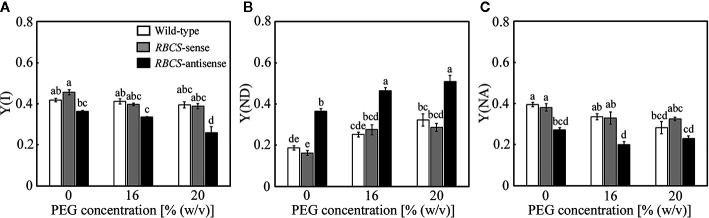
Redox state of P700 after water stress treatment in transgenic rice plants with an increased (*RBCS*-sense) or decreased (*RBCS*-antisense) Rubisco content. Wild-type plants were used as a control. Sixty days after germination, hydroponically grown plants were water-stressed using culture solutions containing PEG at 0, 16, and 20% (w/v) for 2 d under an irradiance of 400–500 μmol photon m^−2^ s^−1^ and day/night air-temperatures of 27/22°C. Y(I) **(A)**, Y(ND) **(B)**, and Y(NA) **(C)** were measured under the conditions of an actinic light intensity of 1200 μmol photon m^−2^ s^−1^, an ambient CO_2_ partial pressure of 40 Pa, leaf temperature of 27°C, and relative humidity of 60–70%. Data are presented as means ± SE (*n* = 4–5). Statistical analysis was carried out using ANOVA followed by the Tukey–Kramer’s test. Columns with the same letter are not significantly different (*p* < 0.05).

Changes in the photochemistry of PSI were examined simultaneously with those of PSII. In wild-type plants, Y(I) tended to marginally decrease in the PEG-treated plants, while slight decreases were also observed in Y(NA) (**Figures 6A, C**). These changes were reflected in increases in Y(ND) (**Figure 6B**), showing that the oxidation of P700 was stimulated by the PEG treatments. In *RBCS*-sense plants, the values of these parameters and their responses to the PEG treatments were similar to those in wild-type plants (**Figures 6A–C**). In *RBCS*-antisense plants, Y(I) and Y(NA) were lower than those in wild-type plants when not treated with PEG (**Figures 6A, C**). These changes were reflected in increases in Y(ND), being 2.0-fold higher than the level in wild-type plants (**Figure 6B**). Thus, the oxidation of P700 was stimulated in *RBCS*-antisense plants without the PEG treatments in the present study, although such a phenomenon was not observed in the previous study (Wada et al., 2018). Y(ND) gradually increased as the (PEG) in the culture solution increased, whereas Y(I) and Y(NA) gradually decreased (**Figures 6A–C**). Y(ND) in *RBCS* antisense plants was higher than that in other genotypes when treated with PEG, showing that P700 in *RBCS*-antisense plants was also in a more oxidized state by the PEG treatments.

Relationships between the parameters of the PET reactions were analyzed (**Table 2**). Data obtained with different genotypes were analyzed together. The mutual relationships between the successive PET reactions were as follows: Y(II) was strongly, negatively correlated with 1−q_L_ and Y(NPQ); 1−q_L_ was strongly, negatively correlated with Y(I), which in turn was strongly, negatively correlated with Y(ND). 1−q_L_ was strongly correlated with these parameters. These results are consistent with those in osmotic-stressed rice plants under normal and high temperatures (Wada et al., 2019). However, some differences were observed compared to the results reported in Wada et al. (2019). In the present study, strong, negative correlations were observed between Y(II) and Y(NPQ) and between Y(ND) and Y(NA).

**Table 2:** Pearson correlation coefficients among the parameters measured in the present study.

	Y(NPQ)	Y(NO)	1-q_L_	Y(I)	Y(ND)	Y(NA)
Y(II)	−0.869***	−0.055	−0.830***	0.893***	−0.967***	0.831***
Y(NPQ)		−0.446**	0.534***	−0.644**	0.847***	−0.843***
Y(NO)			0.424**	−0.316*	0.040	0.196
1-q_L_				−0.876***	0.830***	−0.624***
Y(I)					−0.865***	0.577***
Y(ND)						−0.909***

Data obtained under different conditions of air temperature were analyzed together. *, **, and *** denote statistical significance at p < 0.05, p < 0.01, and p < 0.001, respectively.

The properties in leaf gas-exchange and the photochemistry of PSII and PSI in the PEG-untreated *RBCS*-sense and *RBCS*-antisense plants, and the PEG-treatment response of wild-type plants were basically consistent with those observed in our previous studies (Makino et al., 2000; Hirotsu et al., 2004; Suzuki et al., 2007; Suzuki et al., 2009; Sudo et al., 2014; Wada et al., 2018; Wada et al., 2019).

## Discussion

### Photorespiration Coupled With CO_2_ Assimilation Plays a Crucial Role in the Protection of PSI From Photoinhibition Under PEG-Induced Moderate Osmotic Stress

In the present study, we examined the role of photorespiration coupled with CO_2_ assimilation in the protection of PSI from PEG-induced osmotic stress using Rubisco-transgenic rice plants. The PEG treatments did not significantly affect the relative water content of leaves in all genotypes (**Figure 1**), but substantially decreased *g*_s_ (**Figure 3B**). Stomatal closure is the earliest drought-stress response and was reported to be observed even when water status of plants was unaffected by withdrawal of water (Davies and Zhang, 1991; Chaves et al., 2003). Therefore, moderate osmotic stress was thought to be imposed to the plants by the PEG-treatments used in the present study. In *RBCS*-antisense plants, decreases in the activities of photorespiration and CO_2_ assimilation led to photoinhibition of PSI and PSII under moderate osmotic stress that did not lead to photoinhibition of both photosystems in wild-type plants (**Figures 2**, **3A**, and **4A**). These results clearly indicate that photorespiration coupled with CO_2_ assimilation plays a crucial role in the protection of PSI from photoinhibition under moderate osmotic stress conditions. It has been reported that PSI was more sensitive to drought or osmotic stress than PSII (Huang et al., 2013; Wada et al., 2019). Similar trends were observed in *RBCS*-antisense plants, as PSI suffered from photoinhibition in the 16% PEG-treated plants, whereas PSII did not (**Figure 2**). These results suggest that the weakness of PSI to osmotic stress can be compensated for by the operation of photorespiration coupled with CO_2_ assimilation to some extent.

To examine whether photorespiration contributes the consumption of excess light energy under osmotic stress conditions, elevated CO_2_ condition might be useful as it suppresses photorespiration. However, in the case of *RBCS*-antisense plants, decrease in Rubisco content affect both CO_2_ assimilation and photorespiration. *A* was shown to be limited by Rubisco under elevated CO_2_ conditions where *A* is not limited by Rubisco in wild-type plants (Makino et al., 2000; Suzuki et al., 2009). If osmotic-stress response in *RBCS*-antisense plants was altered under elevated (CO_2_) conditions, it is very difficult to distinguish whether it sorely depended on the suppression of photorespiration.

In contrast, there were no large differences between wild-type and *RBCS*-sense plants in terms of the activities of photorespiration and CO_2_ assimilation, osmotic-stress tolerance, and the photochemistry of PSII and PSI (**Figures 2**–**6**). These results are consistent with those in our previous study, in which these genotypes were exposed to the combination of high irradiance and CO_2_-compensated conditions (Wada et al., 2018). We have suggested that Rubisco was not fully functional in *RBCS*-sense plants The rate of CO_2_ assimilation was not increased proportionally with an increase in Rubisco content in *RBCS*-sense plants, as Rubisco was partially deactivated probably owing to imbalance between the processes of CO_2_ assimilation (Makino and Sage, 2007; Suzuki et al., 2007; Suzuki et al., 2009). The same problem probably arose in the present study.

### Photorespiration Is Possibly Inhibited in *RBCS*-Antisense Plants Under PEG-Induced Osmotic Stress

It has previously been observed that the absolute and/or relative rates of energy consumption by photorespiration increased under drought or osmotic stress in a number of plant species, including rice (Cornic and Briantais, 1991; Haupt-Herting and Fock, 2002; Galmés et al., 2007; Zivcak et al., 2013; Chastain et al., 2014; Wada et al., 2019), accounting for the substantial part of light energy absorbed by leaves. Therefore, it has been suggested that photorespiration plays a role in the consumption of excess light energy, at least in part, under drought or osmotic stress condition. Similar trends were observed in wild-type plants in the present study. Moderate osmotic stress by the PEG treatments tended to increase *J*_PR_/ETRII simultaneously with decreases in *pCi* (**Figures 3C** and **4B**), although the magnitude of the increases were relatively small. However, such trends were not observed in *RBCS*-antisense plants even when *pCi* decreased in the PEG-treated plants. These results could mean that photorespiration was inhibited in *RBCS*-antisense plants under osmotic stress. Our previous report also suggested the inhibition of photorespiration when rice plants were severely osmotic-stressed under high temperature conditions (Wada et al., 2019). These results imply that some processes of the photorespiratory pathway were damaged when excess light energy caused by osmotic stress was far beyond the capacity of photorespiration. As it was reported that the amounts of some photorespiratory enzymes were not affected even under severe drought stress (Wingler et al., 1999), further study is necessary to reveal whether and how photorespiration was inhibited under these osmotic stress conditions. In addition, although *pCi* was higher in *RBCS*-antisense plants than in wild-type plants when not treated with PEG (**Figure 3C**), there were no differences in *J*_PR_/ETRII (**Figure 4B**), which is expected to be higher in wild-type plants owing to the nature of the carboxylase and oxygenase reactions of Rubisco. The method for the evaluation of photorespiration might also need to be improved.

### P700 Oxidation Is Stimulated in Response to PEG-Induced Osmotic Stress Even When the Activities of Photorespiration and CO_2_ Assimilation Are Restricted

It has been reported that the PET reactions responded to drought or osmotic stress in a manner that limits the electron flow toward PSI, leading to P700 oxidation (Golding and Johnson, 2003; Huang et al., 2012; Zivcak et al., 2013; Zivcak et al., 2014; Wada et al., 2019). Consistent with these studies, decreases in Y(II), increases in 1−q_L_, slight decreases in Y(I), and increases in Y(ND) were observed in the present study irrespective of genotype (**Figures 5A, D** and **6A, B**). Decreases in Y(II) in response to osmotic stress were accompanied by induction of NPQ in wild-type plants, *RBCS*-sense plants, and the 16% PEG-treated *RBCS*-antisense plants (**Figure 5C**), as observed in previous studies (Golding and Johnson, 2003; Zhou et al., 2007; Lawlor and Tezara, 2009; Huang et al., 2012; Zivcak et al., 2013; Zivcak et al., 2014; Wada et al., 2019). In the 20% PEG-treated *RBCS*-antisense plants, Y(NO) increased instead of Y(NPQ) (**Figures 5B, C**), as was observed in wild-type rice plants severely osmotic-stressed under high temperature conditions (Wada et al., 2019). These results indicate that the osmotic-stress responses of the PET reactions were normally operative even when the energy consumption by photorespiration and CO_2_ assimilation were largely restricted. PSII photoinhibition occurred in the 20%-PEG treated *RBCS*-antisense plants as *F*_v_/*F*_m_ substantially decreased (**Figure 2A**). This might have restricted the electron flow to PSI and led to P700 oxidation. However, Y(II) was well correlated with Y(I) and Y(ND) when data of the 20%-PEG treated *RBCS*-antisense plants were included in the correlation analysis (**Table 2**). Decreases in *F*_v_/*F*_m_ did also not disturb the relationships among these parameters in osmotic-stressed rice plants, including severely damaged ones under high temperature conditions (Wada et al., 2019). Therefore, electron flow to PSI were likely to be limited by Y(II), not by PSII photoinhibition.

Lumenal acidification is thought to be one of the regulatory factors for the drought-stress responses of the PET reactions as it induces NPQ at PSII (Li et al., 2000; Müller et al., 2001; Huang et al., 2012) and slows down the oxidation of plastoquinol by the cytochrome *b_6_*/*f* complex (Kohzuma et al., 2009; Rott et al., 2011; Tikhonov, 2013; Zivcak et al., 2014; Takagi et al., 2017b). It has also been suggested that over-reduction of the plastoquinone pool suppresses the Q cycle and electron flow at the cytochrome *b*_6_/*f* complex in cyanobacteria (Shaku et al., 2016; Shimakawa et al., 2018). This system was suspected to be operative in osmotic-stressed rice plants (Wada et al., 2019). The PET reactions are thought to be regulated by such processes in response to osmotic stress even in *RBCS*-antisense plants, as 1−q_L_ was strongly correlated with Y(II), Y(NPQ), Y(I), and Y(ND) among the genotypes (**Table 2**).

In the present study, some results were different from those observed in our previous studies. P700 oxidation was not stimulated in *RBCS*-antisense plants in the absence of osmotic stress (Wada et al., 2018). P700 was over-reduced when *RBCS*-antisense plants were exposed to the combination of high irradiance and CO_2_-compensated conditions (Wada et al., 2018), whereas such phenomena as indicated by increases in Y(NA) were not observed in the PEG-treated *RBCS*-antisense plants (**Figure 6C**). The latter can be accounted for, at least partly, by substantial decreases in *pCi* that led to large decreases in energy consumption by photorespiration coupled with CO_2_ assimilation (Wada et al., 2018), whereas the magnitude of the decreases in *pCi* was not as much in the present study (**Figure 3C**). In addition, correlations between Y(II) and Y(NPQ) and between Y(ND) and Y(NA) were not apparent in osmotic-stressed rice plants at normal and high temperatures (Wada et al., 2019). The reason for this discrepancy is unclear. For example, growth conditions were different between these studies as different types of growth chambers were used. Such differences could lead to differences in the responses of the PET reactions, as it was shown that differences in growth irradiance affected the levels of Y(ND) in wheat (Takagi et al., 2019). Recently, Kadota et al. (2019) suggested that excess electron is dissipated by charge recombination within PSI, leading to P700 oxidation. As increases in Y(ND) was observed along with decreases in Y(NA) (**Figures 6B, C** and **Table 2**) when the activity of photorespiration coupled with CO_2_ assimilation was limited (**Figures 3A** and **4A**), charge recombination in PSI might have functioned in P700 oxidation in the *RBCS*-antisense plants used in the present study.

### P700 Oxidation Is Not Sufficient for the Protection of P700 in *RBCS*-Antisense Plants

We have previously reported that PSI suffered from photoinhibition even when P700 was highly oxidized under osmotic stress in rice (Wada et al., 2019). Similar trends were observed in osmotic-stressed *RBCS*-antisense plants (**Figures 2B** and **6B**). These results indicate that P700 oxidation cannot fully protect PSI from photoinhibition under osmotic stress. The reason PSI underwent photoinhibition under conditions of highly oxidized P700 still remains unknown. As PSI-specific photoinhibition has been observed under drought or osmotic stress (Huang et al., 2013; Wada et al., 2019; **Figure 2B**), it is speculated that ROS unavoidably generated within and/or near PSI led to PSI photoinhibition. In addition, P700 oxidation was shown to be gradually stimulated while PSI photoinhibition was induced in *RBCS*-antisense plants by repetitive saturated pulse-illumination under the combination of high irradiance and CO_2_-compensated conditions (Wada et al., 2018), suggesting the possibility that P700 oxidation and ROS generation occurred at the same time.

## Conclusion

In the present study, it is shown that antisense suppression of Rubisco content led to decreases in energy consumption by photorespiration coupled with CO_2_ assimilation under PEG-induced osmotic stress in rice plants, leading to the photoinhibition of PSI and PSII. These results clearly indicate that photorespiration coupled with CO_2_ assimilation plays a crucial role in the protection of PSI from photoinhibition caused by osmotic stress. As PSI was shown to be more sensitive to osmotic stress, photorespiration might compensate for such weakness in PSI. The PET reactions responded to osmotic stress and oxidized P700 in *RBCS*-antisense plants and in the other genotypes. Lumenal acidification and/or the redox state of the plastoquinone pool might primarily regulate the PET reactions under osmotic stress even if the activities of photorespiration and CO_2_ assimilation were restricted. It is shown again that P700 oxidation was not sufficient for the protection of P700 against osmotic stress. ROS unavoidably generated in PSI might damage PSI even if P700 oxidation was stimulated. Overproduction of Rubisco, in contrast, did not alter the activities of photorespiration and CO_2_ assimilation under osmotic stress. As a result, the photochemistry of PSII and PSI were not altered. These results suggest that further modifications of the metabolism of photorespiration and CO_2_ assimilation is necessary to improve drought or osmotic stress tolerance and photosynthesis.

## Data Availability Statement

All datasets generated for this study are included in the article/supplementary material.

## Author Contributions

YS conceived the experimental design. SW performed the experiments. SW and YS analyzed the data. SW and YS wrote the manuscript. SW, CM, AM, and YS edited the manuscript.

## Funding

This study was supported by the Core Research for Environmental Science and Technology (Scientific Research Grant No. AL65D21010 to CM) and Grants-in-Aid for Scientific Research from the Japan Society for the Promotion of Science (No. 18H02111 to YS and No. 16H06379 to AM).

## Conflict of Interest

The authors declare that this research was conducted in the absence of any commercial or financial relationships that could be construed as a potential conflict of interest.

## Abbreviations

*A*, the rate of CO_2_ assimilation; ETRII, the rate of electron trasnport; *g*_s_, stomatal conductance; *J*_PR_, the rate of electron flow donated for photorespiration; NPQ, non-photochemical quenching; P700, the reaction center chlorophyll of photosystem I; *pCi*, an intercellular CO_2_ partial pressure PEG, poly (ethylene glycol); PET, photosynthetic electron transport; PSII, photosystem II; PSI, photosystem I; Q_A_, the primary quinone electron acceptor of photosystem II; 1–q_L_, the fraction of photosystem II centers in closed states; *R*_d_, the rate of respiration under illumination; ROS, reactive oxygen species; Y(II), the quantum efficiency of photosystem II; Y(NO), the quantum yield of non-regulated and non-photochemical energy dissipation at photosystem II; Y(NPQ), the quantum yield of non-photochemical quenching at photosystem II; Y(I), the quantum efficiency of photosystem I; Y(NA), the quantum yield of the acceptor side limitation of photosystem I; Y(ND), the quantum yield of the donor side limitation of photosystem I.
